# Comprehensive characterization of a transgene insertion in a highly repetitive, centromeric region of *Anopheles* mosquitoes

**DOI:** 10.1080/20477724.2022.2100192

**Published:** 2022-07-21

**Authors:** Matteo Vitale, Chiara Leo, Thomas Courty, Nace Kranjc, John B. Connolly, Giulia Morselli, Christopher Bamikole, Roya Elaine Haghighat-Khah, Federica Bernardini, Silke Fuchs

**Affiliations:** a Department of Life Sciences, Imperial College London, London, UK; b Polo d’Innovazione di Genomica, Genetica, e Biologia, Siena, Italy; c Department of Infectious Diseases, King’s College London, London, UK; d Department of Metabolism, Digestion and Reproduction, Imperial College London, London, UK

**Keywords:** Transgene, *PiggyBac*, repetitive region, centromere, whole genome sequencing, fluorescence *in situ* hybridization

## Abstract

The availability of the genomic sequence of the malaria mosquito *Anopheles gambiae* has in recent years sparked the development of transgenic technologies with the potential to be used as novel vector control tools. These technologies rely on genome editing that confer traits able to affect vectorial capacity. This can be achieved by either reducing the mosquito population or by making mosquitoes refractory to the parasite infection. For any genetically modified organism that is regarded for release, molecular characterization of the transgene and flanking sites are essential for their safety assessment and post-release monitoring. Despite great advancements, Whole-Genome Sequencing data are still subject to limitations due to the presence of repetitive and unannotated DNA sequences. Faced with this challenge, we describe a number of techniques that were used to identify the genomic location of a transgene in the male bias mosquito strain Ag(PMB)1 considered for potential field application. While the initial inverse PCR identified the most likely insertion site on Chromosome 3 R 36D, reassessment of the data showed a high repetitiveness in those sequences and multiple genomic locations as potential insertion sites of the transgene. Here we used a combination of DNA sequencing analysis and in-situ hybridization to clearly identify the integration of the transgene in a poorly annotated centromeric region of Chromosome 2 R 19D. This study emphasizes the need for accuracy in sequencing data for the genome of organisms of medical importance such as *Anopheles* mosquitoes and other tools available that can support genomic locations of transgenes.

## Introduction

Advances in molecular technologies, as well as the availability of platforms for Next-Generation Sequencing, have led to an unprecedented investigation of the genome of human disease vector species such as *Anopheles* mosquitoes, vectors of malaria [1–4]. This knowledge has enabled the development of techniques for genome editing and the potential for vector control strategies that aim at mosquito population replacement or suppression [5–7]. Genetic modification requires the integration of a transgene into the genome of the host organism, which confers phenotypes consistent with the control strategy being pursued. Insertions of the transgene can be achieved via a variety of molecular tools; PiggyBac transposons, site-specific recombinases (like AttP/AttB) and CRISPR/Cas9 systems are the most commonly used for genomic engineering applications in insects [6–9]. Whilst AttB/AttP and CRISPR/Cas9 systems allow for a site-directed insertion, PiggyBac-mediated transposon integration occurs semi-randomly into the host genome due to the simple requirement of a TTAA sequence motif. In 2014, the generation of an *Anopheles gambiae* autosomal sex distorter strain gfp124L-2 (renamed Ag(PMB)1 in [10]) was described [11]. This strain carries a modified endonuclease variant, I-PpoIW124L, that cuts ribosomal gene sequences that are highly repetitive and located on the X chromosome of *Anopheles gambiae* mosquitoes [11]. As a result, when expressed during the male spermatogenesis, this endonuclease causes damage to the X-bearing gametes, leaving mostly Y-bearing sperm still functional and generating approximately 95% male offspring. Since only female transmit malaria parasites, this type of autosomal sex ratio distorter alone represents a potential vector control that has been modeled to be more efficient to effectively suppress mosquito populations than the sterile insect technique [12,13]. Moreover, this type of genetic construct has the potential to be developed into a gene drive if placed on the Y chromosome, in this case, all males will inherit the transgene. Therefore, this strain is of interest as a potential vector control but also as an intermediate step toward the development of a self-sustaining gene driven by Target Malaria, which is a not-for-profit research consortium working on developing novel genetic technologies as a tool to fight malaria and to complement existing vector control methods. However, for any strain that is to be considered for release, including for small-scale studies, there needs to be a full and exhaustive characterization of the nature of the transgene insertion.

Ag(PMB)1 was generated via semi-random PiggyBac mediated integration and this strain was selected, based on fertility and male bias values, out of 26 transgenic strains carrying transgenes with various I-PpoI variants [11]. Some of the strains with the same I-PpoI variants, but at different insertion sites, showed a range of male bias and fitness costs, thus indicating a correlation between the phenotype and the insertion site of the transgene. The original insertion site of the Ag(PMB)1 transgene was initially identified on Chromosome 3 R 36D by means of Inverse PCR [11]. Blast results of the flanking sequences at the time however indicated a highly repetitive nature for these elements with multiple hits in the *Anopheles gambiae* genome leaving the possibility of a potential different insertion site. The occurrence of repetitive stretches of DNA makes the process of sequencing, assembly and annotation of genetic elements challenging [14]. Similarly, the integration of a transgene in a region of the genome with highly repetitive sequences can lead to errors in the identification of the integration site. Aware of this possibility, we carried out an extensive analysis to characterize the transgene integration in Ag(PMB)1 further. We combined Whole-Genome Sequencing (WGS), Southern blotting, Fluorescence *in situ* hybridization (FISH) and Polymerase Chain Reaction (PCR) analysis and showed a single integration of the PMB1 transgene with the insertion on Chromosome 2 R 19D.

## Materials and methods

### Mosquito strains

Ag(PMB)1 is a transgenic *Anopheles gambiae* strain where the beta 2 tubulin promoter mediated expression of the I-PpoI structural variant W124L fused to eGFP during spermatogenesis leads to approximately 95% male offspring [11]. The strain was generated by *piggyBac* transposon-mediated integration of the pBac[3xP3-DsRed]b2eGFP::I-PpoI124L transgene into germ cells of *An. gambiae* G3 strain embryos and transgenic individuals can be identified via dsRed marker expression in the eyes and nerve chord. The G3 wild-type is an *Anopheles gambiae/coluzzii* hybrid colony established initially from mosquitoes collected in Gambia in 1975. BF_Ac(PMB)1 is a transgenic PMB1 strain that was introgressed for more than six backcrosses into a local *Anopheles coluzzii* colony collected from Bana, Burkina Faso in 2012. All mosquitoes were maintained under standard mosquito rearing conditions at 27°C ± 1 and 70% ± 5% relative humidity.

### Inverse PCR

Primers were designed to amplify across the 5’ and 3’ genomic insertion boundaries of the Ag(PMB)1 transgene. DNA was extracted from 4 individual pools (containing 5 transgenic larvae each) of Ag(PMB)1 mosquitoes using a Blood and Tissue Kit (Qiagen) following >100 generations of lab maintenance. PCR across the 5’ and 3’ transgene flanking regions was performed using: pB-5SEQ (5′-CGCGCTATTTAGAAAGAGAGA) with genomic primer RH57 (5’-GAAAACCTTACAAGCGTCTTCAA) to amplify the 5′ junction and pB-3SEQ (5′-CGATAAAACACATGCGTCAATT) with RH53 (5’-GATATTATCGCGCTCGGTCC) to amplify the 3′ junction. Each PCR amplicon was Sanger sequenced using both forward and reverse primers and the resulting sequences were aligned to compare genomic flanking and transgenic sequences to the published Inverse PCR data [11].

### Southern blot analysis

A total of 60 individual adult mosquitoes were placed in 3 pools in 1.5 ml low adhesion microtubes and submerged in dry ice until completely frozen, then crushed with a micropestle and incubated at 37°C in a buffer containing 200 µg/ml Proteinase K,200 mM Tris-CL, ph&.5, 30 mM EDTA and 2% SDS. The homogenate was extracted twice in Phenol/Chloroform/Isoamyl alcohol (25:24:1), then incubated with 50 U RNAase A at 37°C for 15 minutes, before being extracted in Chloroform/Isoamyl alcohol (24:1), then precipitated with 3 M Sodium Acetate and Absolute ethanol and resuspended in water overnight. A total of 2.5 µg genomic DNA was digested at 37°C with Fastdigest enzymes at 10 U/µl in reaction times of typically 1 h. Digested genomic DNA was run on 0.8% agarose gels, then transferred to positively charged nylon blots overnight using capillary action. DIG Southern analysis was performed as per manufacturer’s instructions. DIG labeled and unlabeled (control) I-PpoI probes of 499bp were generated with the primers PpoOR: 5’ CTT TGT TGA GGA CCT GCC ACA GT 3’ and PpoIF: 5’ CGA CCT AAG AAG AAG AGG AAG GTG 3’ [15] using the pBac [3xP3-DsRed]b2eGFP::I-PpoI124L plasmid as template DNA using PCR DIG Probe Synthesis Kit (Sigma-Aldrich Catalog number11636090910). Probes were hybridized at 50°C in DIG Easy Hyb buffer overnight and detected using DIG nucleic acid detection kit (Sigma Aldrich cat. No. 11175041910).

### *Sequence library preparation using illumina TruSeq and* in silico *copy number analysis*

DNA extractions were performed on mosquitoes from two strains, namely Ag(PMB)1 and BF_Ac(PMB)1, for each on 10 individuals (5 adult males, 5 non-fed adult females) using a Blood and Tissue Kit (Qiagen). For each sample, 100 ng of input gDNA were sheared using Covaris for a 350 bp insert size. Library preparation was performed using the Illumina TruSeq Nano kit. Each sample was tagged with a unique barcode, followed by three 2 × 150bp High Output V2.5 paired-end sequencing runs on the Illumina NextSeq550 platform, obtaining an average of 265 M reads per sample. Raw data were checked with FastQC software and aligned to *Anopheles gambiae* PEST strain reference genome (AgamP4 from VectorBase) using BWA mem software version 0.7.12. Unmapped reads were *de novo* assembled using SPAdes aligner version 3.13.0 and contigs that included eGFP::I-PpoI124L sequence were aligned with ClustalO webserver and inspected for consistency. To analyze the read coverage of the transgene, sequencing reads were aligned, using BWA mem [16], to AgamP4 reference genome with added expected insertion sequence. This sequence contained the transgene sequences with flanking genomic regions [longer than expected Illumina read of 250 bp) that allowed inspection of reads spanning the putative integration site. The genomic location of the predicted integration site was selected based on AgamP4 PEST reference genome and inverse PCR analysis performed by [11].

**Figure 1.**
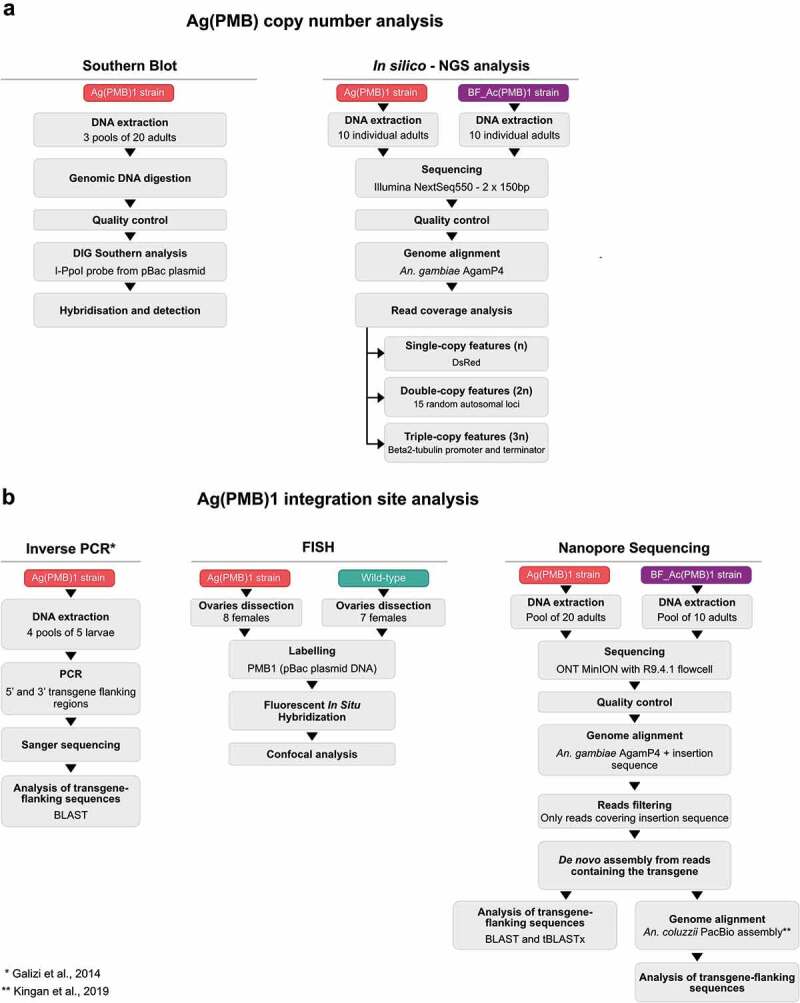
Overview of the methods to determine **A** Ag(PMB1) copy number and **B** Ag(PMB1) transgene integration site.

A custom Python script was written and used to extract informative reads from the alignments and quantify them to obtain the read coverage and copy number of the transgene. Genomic regions used for mean coverage measurements were divided into three separate categories (n-single copy, 2 n-two copies and 3 n-three copies] based on the expected number of copies of a certain genomic feature. As 3 n-three copies feature were selected beta2 tubulin (AGAP008622) promoter and terminator sequences that are expected to be in three copies (two copies from the endogenous gene and a single copy from the transgene). DsRed was selected as n-single copy feature, being present only in the transgene. In addition, arbitrarily selected 15 random 20-bp-long genomic regions on autosomal chromosomes were selected as 2 n-two copies genomic features as these regions should correspond to the coverage on two sets of chromosomes (2 n) (Figure 1A). The random autosomal loci were selected using BEDtools on AgamP4.12 genome annotation [17].

### Nanopore sequencing

The genomic DNA was extracted from a total of 5 BF_Ac(PMB)1 male and 5 female adult mosquitoes, and 20 Ag(PMB)1 female adults using the Blood and Tissue Kit (Qiagen) and prepared for long read sequencing using MinION by Oxford Nanopore Technology (ONT). BF_Ac(PMB)1 DNA was initially fragmented using the Covaris G-tube to obtain fragments of approximately 8kb in length and the ONT adapters ligated (ONT 1D PCR adapters – SQK-LSK109, PBGE12_9066_v109_revM_14Aug2019). Library preparation for Ag(PMB)1 unfragmented DNA was performed according to recommended ONT protocol instructions for both Ligation Sequencing Kit (SQK-LSK109) and Cas9 Sequencing Kit (SQK-CS9109). All samples were purified with AMPure XP magnetic beads. BF_Ac(PMB)1 samples were barcoded and pooled for sequencing onto seven ONT MinION R9.4.1 flowcells. BF_Ac(PMB)1 samples from each library preparation kit were loaded onto separate ONT MinION with R9.4.1 flowcell. The resulted long reads were first processed and quality checked before the alignment to the AgamP4 reference genome concatenated to the insertion sequence (this time without any putative flanking regions) using the Pomoxis tool (https://github.com/nanoporetech/pomoxis) for BF_Ac(PMB)1 samples and minimap2 [18] for Ag(PMB)1 samples. The reads that aligned to the expected insertion region were extracted and *de novo* assembled using the program Canu v1.8 [19].

The assembly was then used to further investigate the insertion site, blasting the regions, adjacent to the transgene, to the PEST reference genome and on VectorBase (https://www.vectorbase.org/). To identify the contig inclusive of the inserted sequence, the *eGFP* and *DsRed* sequences were used as query by BLASTn search [20]. Once the transgenic sequence was detected, both BLAST and tBLASTx searches were performed to better define the genomic region of the insertion. The contig with the transgenic sequences was also realigned to the reference genome with Minimap2 [18] to identify sequence homology between the contig and the reference genome. This latter tool was further used to align the contig inclusive of the transgene sequence to the high-quality assembly obtained for *Anopheles coluzzii* by means of PacBio sequencing [21]. This assembly of PacBio long reads led to a considerably improved resolution of readings in repetitive and unassembled regions in the *An. gambiae* PEST reference. In particular, Kingan et al. were able to assign 40% of sequences that so far were misplaced in the artificial AgamP4 PEST chromosome UNKN to specific chromosomes. Since many of the BLAST hits were reported in the UNKN chromosome, the PacBio *An. coluzzii* assembly was used in the present analysis to assign the potential insertion site (Figure 1B). A dot plot was produced to compare the PacBio *An. coluzzii* assembly and the AgamP4 PEST reference genome and visualize the putative position of the inserted sequence. The dot plot was generated using the online tool D-Geneies v1.2.0 (run ID: PNqgs_20210713201144) [22].

### *In* Situ *Hybridization of polytene chromosomes*

Ovaries at Christopher stage III from 18/33-h half-gravid females were dissected from 7 wild-type and 8 Ag(PMB1) individuals and fixed for 24-h at Room Temperature (RT) in Carnoy’s solution. Polytene chromosome preparations were obtained according to Xia et al. [23]. Chromosome spread quality check was performed using a phase-contrast microscope and then immersed in Liquid Nitrogen. Subsequently, they were dehydrated in a series of ethanol (50%, 70%, 90%, 100%) for 5 minutes each and then allowed to air dry at RT and stored at – 20°C. To perform *Fluorescent In Situ* Hybridization (FISH) on each chromosome preparation, 800 ng of pBac [3xP3-DsRed]b2eGFP::I-PpoI124L plasmid DNA [11] was labeled with dUTP-Cy5 (GE Healthcare) by nick translation reaction (ROCHE). The reaction mixture was incubated at 15°C for 90 minutes and stopped by heating at 65°C for 10 minutes. PCR reaction (F-TATCGGCTGCAACATCAAAC, R-ACAGAGGTTGTTGAGGAACCA) was used to generate probes from the gene AGAP004670 [24]. DNA probes were precipitated by adding 1/10 volume of 3 M NaAC and 2.5 volume of 100% ethanol and centrifuged at 14,000 g for 20 minutes at 4°C. The Probe was then resuspended in hybridization buffer that was pre-warmed at 37°C. FISH was performed as previously described by Xia et al. [23].

After denaturation, hybridization, and washing steps, the microscope slides were mounted with Prolong Antifade + DAPI, sealed with Cytobond and stored at 4°C until usage. Microscope slides were analyzed using a Leica SP8 inverted confocal microscope. Pictures were observed with a 40x oil immersion objective and analyzed using Fiji and Photoshop.

## Results

### Characterization of the PMB1 integration flanking sites by inverse PCR

**Figure 2.**
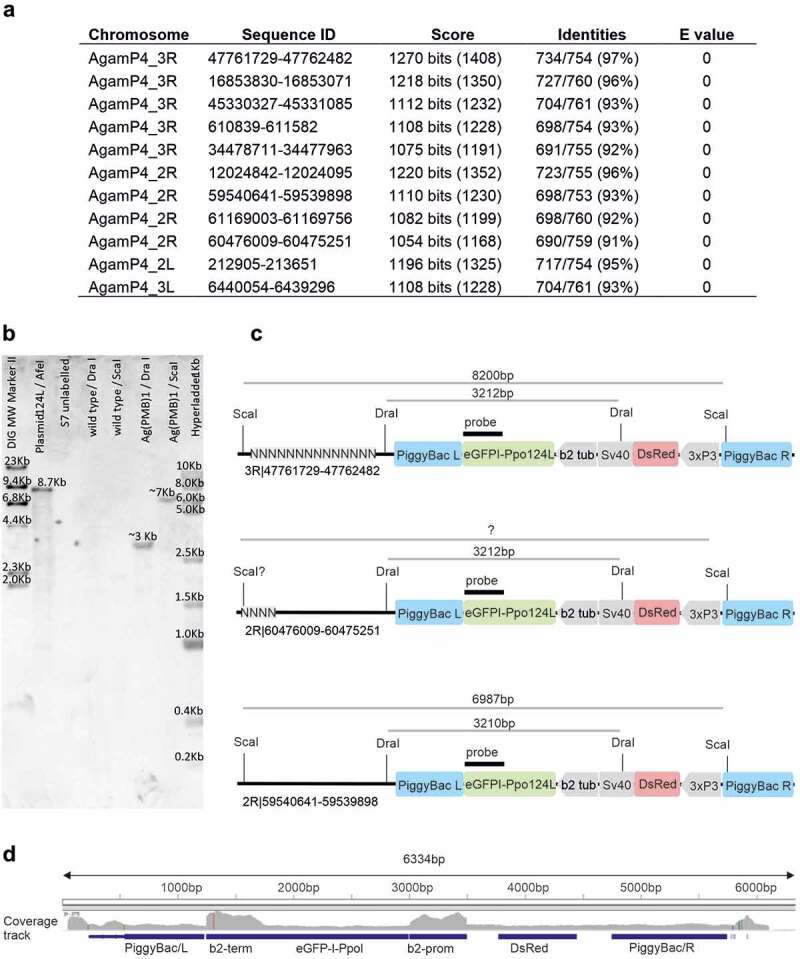
Potential chromosomal insertion sites of the Ag(PMB)1 transgene based on Inverse PCR and subsequent blast search in Vectorbase database. **A** A blast search of the sequence obtained by Inverse PCR revealed 12 autosomal locations with high sequence similarity (>90%) over long Inverse PCR sequence stretches (~700/788bp), including the Chr 3 R 36D location (3 R|47761729-47762482) previously proposed as the insertion site [11]. **B** Southern blot probed with *I-PpoI* specific transgene sequences. As expected, a 8.7kb band was detected in ‘Plasmid’ positive control consisiting of pBac [3xP3-DsRed]b2eGFP::I-PpoI124L linearized with AfeI. No signals were detected in DraI-cut and ScaI-cut wild type genomic DNA. Single postive bands of the expected sizes, around 3 and 7kb, were detected in Ag(PMB)1 genomic DNA cut with DraI and ScaI. respectively. All numbers refer to DNA fragement sizes in kb. **C** Schematic maps of transgene insertion in Ag(PMB)1 at the Chromosome 3 R 36D and two newly identified potential integration sites on Chromosome 2 R in the *Anopheles gambiae* PEST reference genome (source Vectorbase: AgamPEST). Digestion of Ag(PMB)1 genomic DNA with DraI and ScaI is expected to produce 3.2 and 7–8.2kb fragments respectively, both of which can be detected using a probe against I-PpoI (in green). **D** Illumina sequencing reads aligned to the transgenic sequences and integration site flanking regions.

In order to characterize the integration site of the transgene in the Ag(PMB)1 strain we first performed an inverse PCR using primers previously used by Galizi et al [11]. Our results showed the same flanking regions (~400 bp on each side) as originally described for this strain (**Supplementary Figure 1**). A subsequent BLAST search of the combined 5’ and 3’ flanking sequences found the best match with over 97% sequence identity over a 754-bp alignment length at the previously identified Chromosome 3 R 36D location. However, we also obtained over 500 hits on different chromosomal locations, suggesting that the integration occurred in a very repetitive region in the genome and that the integration potentially occurred at a different site than previously described. In fact, 12 of these autosomal hits showed over 90% sequence identity of an alignment length of over 700 bp and they also contained the TTAA recognition sites required for piggyBac mediated insertion of the transformation plasmid (Figure 2A).

### Confirmation of a single integration event by southern blot and illumina sequencing

In order to determine whether the integration could have occurred at multiple repetitive sites or whether there may have been copy number or structural alterations in the transgene, we performed a Southern blot analysis. Our results revealed only single bands produced by two different enzyme digestions of Ag(PMB)1 genomic DNA, demonstrating that Ag(PMB)1 indeed contains a single insertion of the transgene (Figure 2B). Similar to our previous BLAST search of sequences obtained by Inverse PCR, the size of the DNA fragment spanning part of the transgene and its flanking sites could not be matched to a specific insertion site. This was due to multiple possible sites with similar fragment sizes and the fact that some possible sites were not assigned to chromosomes in the *Anopheles gambiae* PEST reference genome (Figure 2C). In addition, we investigated whether Illumina sequencing could be used instead of Southern blot analysis to confirm transgene integration events. We aligned WGS reads to a sequence of the transgene flanked by genomic regions upstream and downstream from the putative integration site. The analysis of the aligned reads showed that, as expected for a single integration, the coverage on the loci specific to eGFP::I-PpoI124L transgene and *piggyBac* flanking arms was consistent with single copy coverage (n) compared to the coverage from autosomal (2 n) loci as the heterozygous individuals only contained one copy of the transgene (1 n). In addition, the coverage at the promoter and terminator regions of endogenous and transgenic beta-tubulin gene was higher and consistent with 3 n (expected single copy of the sequence from the transgene and two copies from the endogenous sequences) (Figure 2D). The analysis also showed inconsistently high coverage in the immediate upstream and downstream transgene-flanking regions. However, this observation confirms the repetitive nature of the sequences flanking the integration site, which was also confirmed with BLAST search that resulted in more than 500 hits of homologous sequences from other sites in the *Anopheles* genome. Overall, this type of coverage analysis could be a useful alternative to Southern blot analysis to confirm copy number when characterizing transgene insertions.

### Limitations of whole genome sequencing approaches to pinpoint integration sites in insufficiently annotated regions in the reference genome

Because the transgene flanking regions occur in a highly repetitive region, which are usually collapsed into a single sequence in de novo assemblies using Illumina reads, we performed Nanopore sequencing analysis to obtain longer reads that would span this region. Despite low coverage (<10x) we obtained 3’ end sequences that significantly aligned to the PMB1 transgene and to a 563bp region about 1.5 kb upstream of the repetitive sequence corresponding to a locus on chromosome arm 2 R (2 R | 58,941,996–58,942,559). This places Ag(PMB)1 in band 19D of chromosome arm 2 R, a region previously identified as heterochromatic and rich in retroelements and tandem repeats [25]. Some reads also aligned the transgene flanking regions on the 3’ end to the 3 R (3 R | 47,762,128–47,762,483) which corresponds to the published insertion site at 36D of chromosome 3 R [11] although this alignment was much more tenuous with lower mapping quality compared to the alignment on the 2 R band 19D region. This suggests the previously published insertion site of chromosome 3 R band 36D was prematurely attributed due to an inability to characterize a sufficient length of the flanking regions.

**Figure 3.**
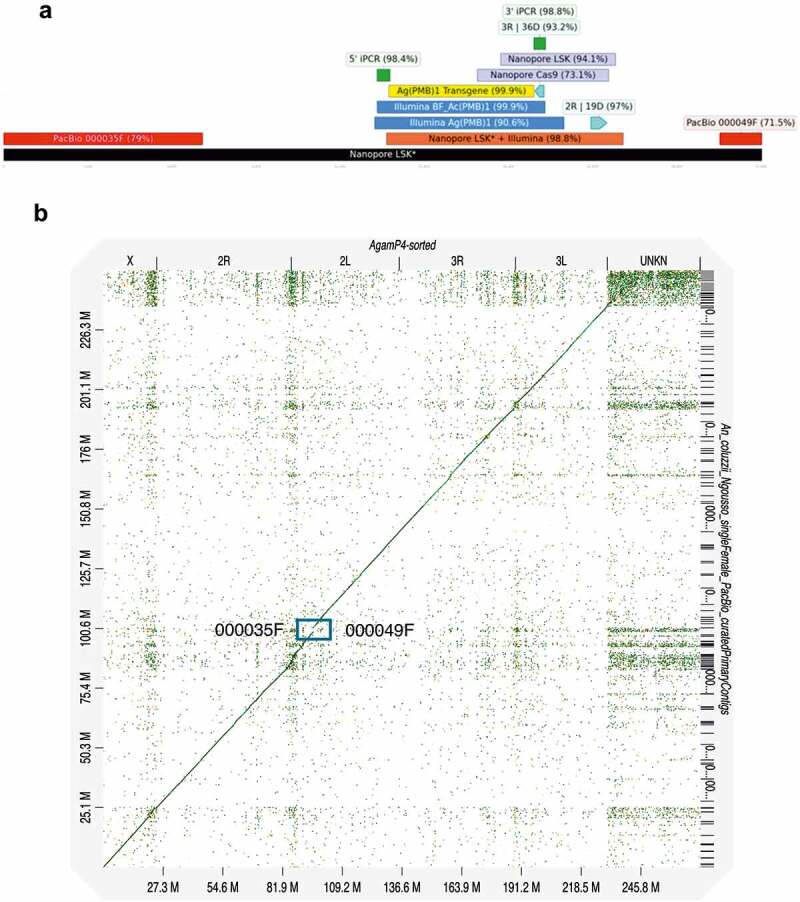
WGS sequencing analysis of the PMB1 insertion site. **A** Alignment of the PMB1 transgene and flanking sequences from Inverse PCR (iPCR) from Ag(PMB)1 (New and [11]), Illumina sequencing from Ag(PMB)1 and BF_Ac(PMB)1, Nanopore sequencing LSK* from BF_Ac(PMB)1), Nanopore Cas9 and Nanopore LSK from Ag(PMB)1. **B** Dot plot of the alignment between the de novo assembly of An. coluzzii genome (contigs on the y axis) and the AgamP4 PEST reference genome (on the x axis). The box shows the genomic position of the PacBio contigs (000035 F and 000049 F), where there was an alignment of the transgene flanking regions.

We also investigated the transgene location in a different genetic background. A *de novo* genome assembly was performed using nanopore sequencing on the introgressed BF_Ac(PMB)1 strain that contains the same transgene but is located in an *Anopheles coluzzii* instead of an *An. gambiae* background. Assembly of reads with transgenic sequences (DsRed and eGFP::PpoI124L) resulted in a 27 kb long contig, which showed high sequence identity (> 90%) when compared to the sequences obtained with inverse PCR and Illumina sequencing from Ag(PMB)1 and BF_Ac(PMB)1 (Figure 3A). This result supports correct assembly of the reads obtained from nanopore sequencing. Additionally, the comparison of the transgene-flanking regions from the transgene bearing contig with high-quality *de novo* assembly of *An. coluzzii* [21] showed high sequence identity with two *An. coluzzii* contigs (000049 F and 000035 F). A 17.6 kb DNA sequence from 5’ transgene-flanking region and a 2.6 kb sequence from the 3’ transgene-flanking region matched *An. coluzzii* contigs 000049 F and 000035 F, respectively, with relatively high sequence identity (> 70%). According to the dot plot, both *An. coluzzii* contigs (000049 F and 000035 F) correspond to the centromeric region of the 2 L chromosome in proximity to the 2 R centromeric chromosome region predicted by the nanopore sequencing results of Ag(PMB)1 (Figure 3B). Despite lower sequence identity of the immediate repetitive sequences neighboring the transgene, the long flanking regions around the transgene on the 27 kb contig allowed us to locate the transgene more precisely compared to the results from other sequencing methods. Importantly, the 27 kb contig provided an accurate consensus sequence of the inserted transgene and its genomic context.

### In situ *hybridization as a tool to locate transgenes in highly repetitive non-annotated regions*

**Figure 4.**
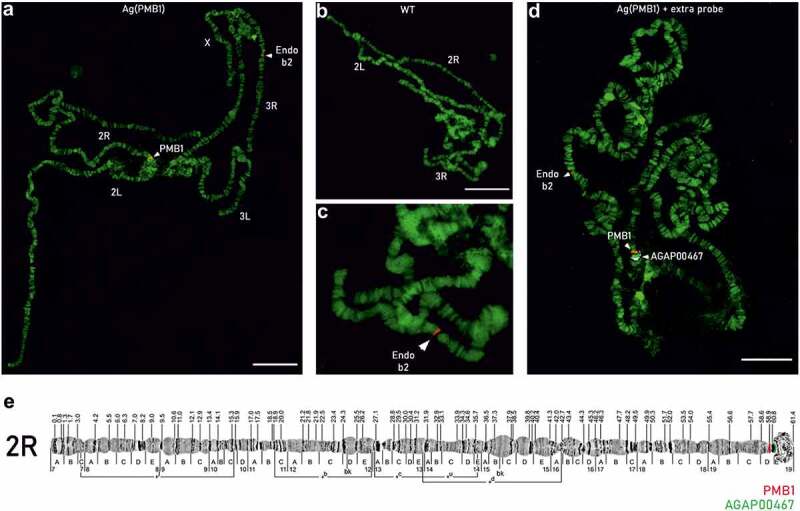
FISH analysis on polytene chromosomes of Ag(PMB)1 and wild-type (WT) mosquitoes. FISH was performed with a probe consisting of the original transformation plasmid specific to the transgene in Ag(PMB)1 individuals as well as the endogenous beta2 tubulin promoter (AGAP008622) on Chromosome 3 R:13,725,120 … 13,726,724 in WT and Ag(PMB)1 individuals **A** PMB1 samples showed two red signals labeling the endogenous beta2-tubulin promoter on chromosome 3 R and the PMB1 transgene insertion site in the proximity of the centromere of chromosome 2 R. **B** and **C** WT samples only showed one probe signal for the endogenous beta2 tubulin promoter in the distal region of chromosome 3 R **D** Signals from the PMB1 transgene (in red) and the flag gene AGAP004670 with known integration site (in white). **E** Chromosomal map of the proposed location of the PMB1 transgene and the reference gene (AGAP004670) with known chromosomal location at Chromosome 2 R: 60845862 … 60895998.

In order to overcome the limitations of the various sequencing approaches and existing reference genomes we then applied FISH to validate the potential centromeric 2 L/2 R chromosomal insertion site of the transgene. This technique has been widely used on polytene chromosomes for the creation of cytogenetic maps and genome annotation in *Anopheles* mosquitoes [26,27]. A probe consisting of the original transformation plasmid was generated to bind the transgene in Ag(PMB)1 individuals as well as the endogenous beta2 tubulin promoter (AGAP008622) on chromosome 3 R:13,725,120.13,726,724 as endogenous positive control for Ag(PMB)1 and wild-type (WT) individuals. In total, we examined 54 polytene chromosomes from 7 WT *An. gambiae* individuals and 63 polytene chromosome from 8 Ag(PMB)1 individuals. As expected for a single integration of the transgene, 94% of PMB1 individuals (59 out of 63) showed two FISH signals, one corresponding to the endogenous beta2-tubulin promoter, located on the chromosome 3 R, band 31 division A and the other one to the transgene in the chromocenter of chromosome 2 R band 19, division D-E (Figure 4A). A few samples (n = 4) showed only one signal in the chromocenter of chromosome 2 R but none in chromosome 3 R. As expected, 70% of wild-type samples (38 of 54) showed only one single fluorescent band that corresponded to the endogenous beta2 tubulin promoter on chromosome 3 R (Figure 4B and Figure 4C), the rest (n = 16) showed no fluorescent signal. The failure of labeling of the endogenous site on chromosome 3 R for WT and PMB1 samples, was possibly due to insufficient amount of the probe in these samples. Importantly none of the 54 WT samples showed a signal on chromosome 2 R. To confirm the transgene site integration on chromosome 2 R, band 19D, a second probe targeting exon 1 of a gene, AGAP004670, with known location on chromosome 2 R, band 19, division E (position 60845862–60895998) was used for FISH analysis. As expected, the probe (in white) gave a signal in between the chromocenter of chromosome 2 R and the signal from the PMB1 transgene (in red) (Figure 4D).

Together, these results confirmed that the insertion of the PMB1 transgene is on chromosome 2 R 19D (Figure 4E).

## Discussion

In the last 20 years, progress in the development of genetic strategies for the control of vector-borne diseases such as malaria has grown in parallel with the exponential availability of whole-genome sequencing data [1–4,21,28,29]. Although Next-Generation Sequencing technologies have provided reliable sequence information for most of the genome, heterochromatic and repetitive regions can often lead to uncertainty or errors in DNA assemblies. It has been estimated that 33% (about 86 Mbp) of the *An. gambiae* genome is composed of repetitive elements, which are mostly found in the pericentromeric regions of the autosomes and make up almost the entirety of the Y chromosome [28,30]. A substantial effort has been made by the scientific community to improve data on genome assembly and annotation over the last years [1–4,21,28,29,31]. In a study by Sharakhova et al. [31] cDNA, BAC clones and PCR amplified gene-fragments were used as probes for in situ hybridization to place about 33% of previously unmapped sequences that in total, accounted for around 15 Mbp. These sequences fell mostly within pericentromeric regions of the chromosomes; in addition, around 1.32 Mbp covered the physical gaps between scaffolds on euchromatic parts of the chromosomes. Further, in 2019, a new low-input PacBio-based approach led to a de novo assembly from a single *An. coluzzii Ngousso*; this study succeeded in placing 667 (>90%) of previously unplaced genes into their appropriate chromosomal contexts in the AgamP4 PEST reference genome [21]. AgamP4 still includes a number of unplaced contigs (27.3 Mb excluding unidentified nucleotides -Ns-) that are currently labeled as the ‘UNKN’ (unknown) chromosome as well as misassembled haplotype scaffolds. Further, over 6302 gaps of Ns (ranging from 20bp-36kb) in the primary chromosome scaffolds makes the areas of genome assembly still highly fragmented [21].

In this manuscript, we report a number of strategies based on sequencing data as well as Southern blotting and FISH analysis that were used to characterize transgene site integration in the *An. gambiae* strain Ag(PMB)1. This strain produces a male bias phenotype that is desirable for potential vector control. As part of the strain assessment a molecular characterization of its transgene and insertion site was performed [32].

Here, we show that the preliminary assignment of the transgene location to Chromosome 3 R 36D [11] was incorrect due the presence of highly repetitive sequences at the insertion site. Inverse PCR is a standard method that allows the isolation and sequencing of the regions flanking the transgene. However, in our case, a BLAST analysis provided 500 hits of which 12 showed 90% sequence identity over almost all of the flanking sequence and they also contained the TTAA recognition sites required for PiggyBac mediated insertion. Based on these results, we concluded that Inverse PCR, which produces relatively short flanking sequences, is less suitable for highly repetitive long sequences (>700 bp) to ascertain correct identification of transgene landing sites. We then ruled out the possibility of multiple insertions of the transgene in the genome by performing Southern blotting and coverage analysis based on Illumina sequencing data generated using samples of the Ag(PMB)1 strain. Both approaches confirmed a single insertion of the transgene and coverage analysis could be used as an alternative to classical Southern blot analysis to determine copy number of transgenes. However, neither provided definite evidence of the location of the transgene. Nanopore sequencing was then used to generate long reads of genomic sequence with the aim of extending the sequence obtained in the regions flanking the transgene. These data were used for a BLAST analysis that suggested band 19D of chromosome 2 R as the most likely location of the transgene, however some reads also aligned to band 36D on chromosome 3 R, leading to persistent ambiguity of the precise genomic location of the transgene. Alignment of Nanopore sequencing data generated from the introgressed *An. coluzziii* BF_Ac(PMB)1 strain to the *de novo An. coluzziii* Ngousso assembly [21] was also unsuccessful in determining the exact insertion site of the transgene.

Finally, we examined the presence of the transgene in the genome of the Ag(PMB)1 strain by FISH analysis. A fluorescent probe consisting of the original transformation plasmid was generated that was complementary to and could therefore hybridize with the sequences of the PMB1 transgene on polytene chromosomes preparations. The result was consistent with one of the integration sites identified by sequencing-based approaches and show that FISH can be used as a complementary tool to confirm insertion sites in highly repetitive regions.

However, genome assembly for species with highly polymorphic genomes, such as mosquitoes, can be challenging, novel techniques in sequencing and scaffolding methods promise to greatly improve current data that could support the development of novel targets for vector control, off-target analysis, and characterization of insertion sites of transgenic strains. In combination with phenotypic data, this will support the prediction of dynamics of a transgene in potential target field populations.

## Supplementary Material

Supplemental Material

## Data Availability

The data that support the findings of this study will be openly available at https://zenodo.org/record/6010699 (DOI: 10.5281/zenodo.6010699) upon publication.
