# Supplementary figures and images for: Comprehensive characterization of a transgene insertion in a highly repetitive, centromeric region of Anopheles mosquitoes
Source: Pathog Glob Health. 2022 Jul 21;117(3):273–83. doi: 10.1080/20477724.2022.2100192 (PMC10081084; doi:10.1080/20477724.2022.2100192)

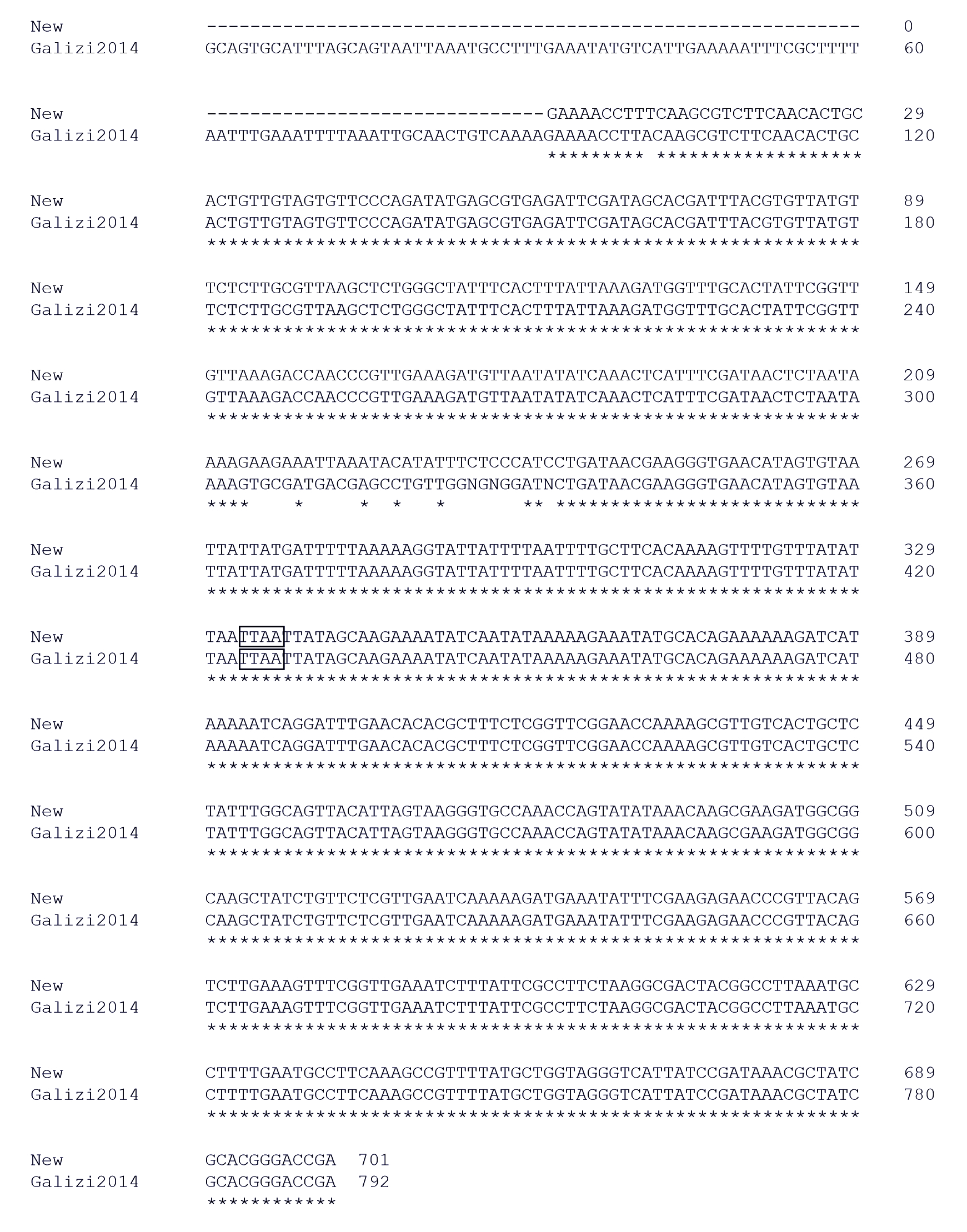

Supplement: Supplemental Material [file YPGH_A_2100192_SM8197.jpg]
